# Ultrafast Studies of ZrTe_3_ by Transient Absorption Spectrometer

**DOI:** 10.3390/ma15155420

**Published:** 2022-08-05

**Authors:** Shakeel Ahmed, Wang Rui, Faizah Altaf, Jahanzeb Khan, Patrizia Bocchetta, Han Zhang

**Affiliations:** 1 College of Physics and Optoelectronic Engineering, Shenzhen University, Shenzhen 518060, China; 2 State Key Laboratory for Mesoscopic Physics and Frontiers Science Center for Nano-Optoelectronics, School of Physics, Peking University, Beijing 100871, China; 3 Department of Environmental Sciences, Women University of Azad Jammu & Kashmir, Bagh 12500, Pakistan; 4 School of Materials Science and Engineering, Georgia Institute of Technology, North Avenue, Atlanta, GA 30332, USA; 5 Department of Chemistry, University of Azad Jammu & Kashmir, Muzaffarabad 13100, Pakistan; 6 Dipartimento di Ingegneria dell’Innovazione, Università del Salento, via Monteroni, 73100 Lecce, Italy

**Keywords:** transient absorption spectrometer, ZrTe_3_ nanosheets, excitons

## Abstract

Two-dimensional (2D) tri-TMDCs carrier dynamics provide a platform for studying excitons through Ultrafast Pump-Probe Transient Absorption Spectroscopy. Here we studied the ZrTe_3_ nanosheets (NTs) exciton dynamics by transient absorption (TA) spectrometer. We observed different carrier dynamics in the ZrTe_3_ NTs sample at different pump powers and with many wavelengths in the transient absorption spectrometer. The shorter life decay constant is associated with electron-phonon relaxation. Similarly, the longer-life decay constant represents the long live process that is associated with charge separation. The interactions between carrier-phonons at nanoscale materials can be changed by phonons quantum confinements. The hot carrier lifetime determined the strength of carrier phonon interactions. The value of fast decay in the conduction band is due to carrier relaxation or the carrier gets trapped due to surface states or localized defects. The value of slow decay is due to the recombination of surface state and localized defects processes. The lifetime declines for long wavelengths as size decreases. Whereas, during short wavelength-independent decay, carrier characteristics have been observed. TA spectroscopy is employed to investigate insight information of the carrier’s dynamical processes such as carrier lifetime, cooling dynamics, carrier diffusion, and carrier excitations. The absorption enhanced along excitons density with the increase of pump power, which caused a greater number of carriers in the excited state than in the ground state. The TA signals consist of trap carriers and (electron-hole) constituents, which can be increased by TA changes that rely on photoexcitation and carrier properties.

## 1. Introduction

Two-dimensional TMTCs possess exceptional electronic and optical properties and have potential applications in many optoelectronic devices. TMTCs semiconducting chalcogenides TX_2_, (where T = W or Mo and X = Se or S) are used in photodetectors, transistors, flexible substrates in integrated circuits, and in some other device applications [1]. Moreover, transition metals 2D tri-TMTCs like TX_3_ (where T = Zr, Ti, Nb, Ta, Hf or Ta and X = Te, S or Se) are rarely studied compared to TX_2_TMTCs. The current studies on these materials showed potential applications in the field of flexible electronics, optoelectronics, nano-electronics, third-generation, novel nanophotonic devices, and solar photovoltaics. Due to the excellent optical properties and potential applications, the use of 2D materials in the current industry is increasing day by day. Despite of huge research on 2D materials still, comprehensive studies are required [2,3,4].

The ZrTe_3_ (zirconium compound) was first discovered in the 1960s. It got huge attention due to charge density wave (CDW) as a bulk material with transitions of CWD at a temperature of ~63 K and has the transition of superconductivity at about 2 K [5,6], which shows that ZrTe_3_ still needs systematic and comprehensive investigation. ZrTe_3_ is one of the prototypical CDW materials, which possess specific heat signatures [7], resistivity, and Hall coefficient variances [8]. Moreover, periodic lattice distortions (PLD) have been examined in soft phonon mode phase transition [9]. Electron-phonon coupling and variations in Raman active phonon modes in CDW transitions have been observed [10]. Moreover, the TCDW band gap has been observed in ZrTe_3_ by photo-emission spectroscopy [11].

Both angle-resolved photoemission spectroscopy observations and the electronic band calculations of ZrTe_3_ have Fermi surface of quasi 1D and 3D in electron sheets and hole sheets [12,13]. At the initial stage, it has been observed that metallic ZrTe_3_ monolayers exhibit bi-axial tensile strains, where the bandgap increased from (0.1–0.52) eV by extending the strain from 4% to 8% [14]. It has been also observed that applied pressure of about 2 GPa can make it more metallic by a change in the Fermi surface. Whereas, increasing the pressure up to several GPa makes it a typical semiconductor or semimetal due to the enhancement in the Seebeck coefficient. Moreover, it has been observed that 2D TMTCs band gap values depend upon the van der Waals forces and the covalent bond between the adjacent layers [15,16,17], which shows that electronic properties are sturdy chalcogenide dependent. Moreover, it has been examined that energy gap values change from (0–0.1) eV for ZrTe_3_−ZrSe_3_, which represents a novel class of slight bandgap semiconductors with fascinating physical properties. E.g., like ZrTe_3_ and some of them have thermoelectric-based potential applications.

The ZrTe_3_ electronic band structure is calculated through angle-resolved photo-emission spectroscopy (ARPES). From ARPES measurements, it is observed that ZrTe_3_ Fermi surface contains a 1-D electron sheet and 3-D hole sheet. Moreover, from thermopower data it is seen that ZrTe_3_ monolayers band gap (Eg) vary from 0.1 eV to 0.52 eV by applying pressure up to 2 GP. Moreover, the Seebeck coefficient values are much higher compared to typical semiconductors or semi metals. The TMTCs band gap values mainly depend upon two factors: interlayer covalent bond interactions and van der Waals forces. This indicate that electronic and optical properties of these TMTCs rely on chalcogen. The ZrTe_3_ solid solution has an energy gap from 0–1.1 eV. These properties present a novel class of semiconductors with a narrow bandgap, which shows excellent physical properties. E.g., predominant ZrTe_3_ may have potential for thermoelectric applications [18].

We carry out a detailed ultrafast study and optical investigation of the photoexcited carriers and excitons dynamics in ZrTe_3_ NTs using an ultrafast TA spectrometer. In TA spectra, we observed the excitons absorption peak and strong e-e (electron-electron) interactions in ZrTe_3_ NTs. These important excitonic effects have also been examined in the band structures of TMDCs in both ground and photo-excited states [18].

## 2. Materials and Methods

### 2.1. Ultrafast Transient Absorption Spectrometer Setup

**Figure 1:**
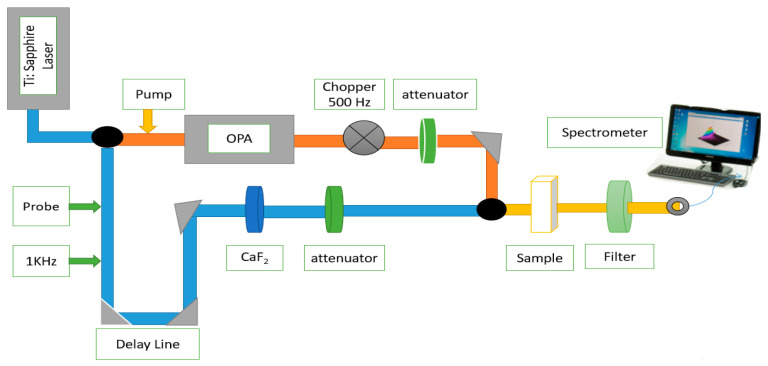
Systematic diagram of ultrafast transient absorption spectrometer setup.

The TA spectrometer has the approximately same mechanism as stimulated emission microscopy which obtained information from the sample at an excited state. The TA spectrometer can give high time-resolved detailed absorption spectrum information, which is an important tool for studying energy level structure and energy relaxation or charge transfer of excited state. A general setup for ultrafast pump-probe TA spectroscopy consists of two laser pulses, which overlap at the desired sample placed before the detector, as shown in Figure 1. The purpose of the pump pulse is to excite the sample and a weak probe pulse falls at the photo-excited sample with a time delay ‘τ’ after the pump pulse. These two consecutive probe pulses caused absorption, one in the absence and the second in the presence of the pump pulse, which has been used to observe the pump-induced absorption alteration in the sample at the delay time τ, i.e., ΔA(τ). Furthermore, a spectrally broad probe pulse has been utilized, absorption (∆A) as a function of delay time (τ) and probe wavelength (λ) i.e., ΔA (λ, τ) is recorded. The photo-induced dynamic processes are important in studying functional materials, such as excited-state energy, intersystem crossing, refs. [19,20] transfer of electron and hole [21], and photoisomerization [22]. Here, we examined the ZrTe_3_ NTS carrier dynamics by using an ultrafast TA spectrometer [23].

#### ZrTe_3_ Preparation Method

The ZeTe_3_ solution is put into the 1 mm cuvette as a sample to be tested. In an ultrafast TA setup, a pump wavelength of 400 nm with different pump powers (0.1, 0.2, 0.4, 0.6, 0.8) mW and probe wavelength at 480 – 770 nm is used. All the TA experiments are performed at room temperature. To prepare ZrTe_3_ NTs (having nanoparticles of different sizes), we employed the liquid-phase exfoliation (LPE) method. Generally, probe and bath sonication methods were used to prepare ZrTe_3_ NTs with the help of centrifugations. Firstly, grounded the 100 mg ZrTe_3_ crystal for four hours to convert the crystal into powder form with 100 mL NMP. After grinding, the ZrTe_3_-NMP suspension with a concentration of 1 mg/mL was probed and sonicated for 12 h. During these processes, the van der Waals interaction between the layers of bulk ZrTe_3_ could be easily broken and the liquid environment of NMP can further expand the interspace of molecular layers. The ZrTe_3_ NTs can be obtained ultimately by extracting supernatant in obtained ZrTe_3_/NMP suspension after centrifugation at 3000 rounds per minute (rpm) for 3 min.

## 3. Results

**Figure 2:**
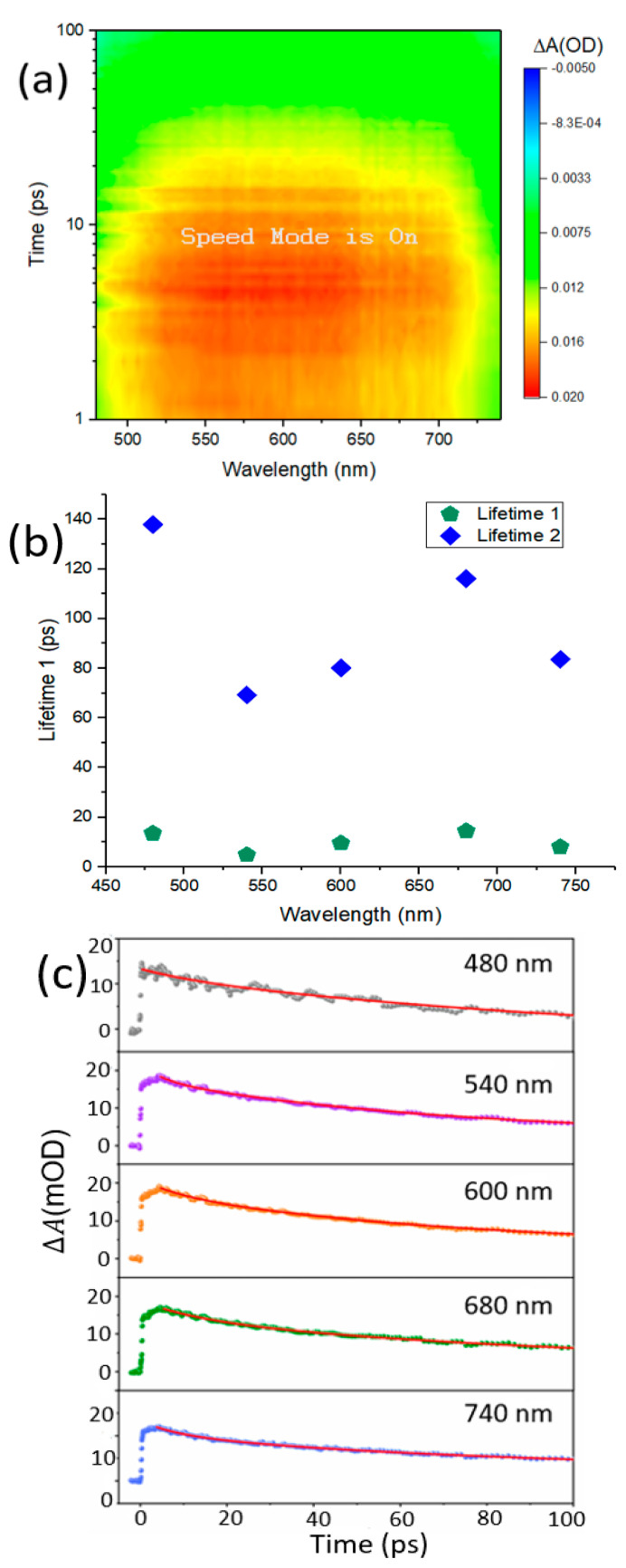
(**a**) Transient absorption spectra of ZrTe_3_ NTs sample recorded at a pump pulse of 400 nm having pump power of 0.8 Mw (**a**) 3D plot of the transient absorption spectra. (**b**) Shows the relationship between lifetime (ps) and wavelength (nm) associated with electron-phonon relaxation. (**c**) Fitting results of the decay curves of ZrTe_3_ for various probe wavelengths have been drawn between Transient Absorption (∆A) Vs pump-probe delay time (τ) for ZrTe_3_ NTs. The fitting results may have a 10% error from the standard deviation.

In this work, a ZrTe_3_ NTs sample has been investigated temporally and spectrally using a TA spectrometer. For all measurements, the pump wavelength was used at 400 nm which is much higher compared to the probe light, at a different pump power, and with different probe wavelengths. Figure 2 demonstrates the differential absorption signal over a small-time scale using a pump pulse of about 3.1 eV (400 nm). The values of the different probe wavelengths are labeled in Figure 2. After pump excitation, the signal gets a peak value limited by the instrumental response and decays rapidly around 10 ps. This quick decay activity is the main topic of this research paper.

The transient absorption (TA) spectra are examined at a pump pulse of 400 nm having pump power of 0.8 Mw representing the excitons dynamics, as shown in Figure 2a. By laser excitation photo charge carriers generated from hot excitement, these hot excitons recline from higher excited states (the high-energy levels) to the lowermost excited state, also known as the band-edge state. Then finally they go back to the ground state by surface trapped defects or through non-radiative and radiative processes in the ZrTe_3_ NTS sample. Figure 2a demonstrates the 3D plot of TA spectra examined from the ZrTe_3_ sample. These results have quite a resemblance with reference [24]. The red color in the Figure 2a shows the excitons, which is the solid proof that excitons exist in ZrTe_3_ materials. In Figure 2b, a plot has been drawn between wavelength and lifetime, which shows a nonlinear behavior. The shorter lifetime or decay constant is associated with electron-phonon relaxation, which is shown by the green color. Similarly, the long lifetime or decay constant represents the long live process that is associated with charge separation, which is shown by the blue color in Figure 2b. Generally, carrier-impurities and carrier-phonons scattering is the main cause for limiting the carrier mobility in bulk type semiconductors. The interactions between carrier-phonons at nanoscale materials can be altered by phonons quantum confinements. The hot carrier lifetime determined the strength of carrier phonon interactions, this carrier phonon interaction for ZrTe_3_ NTS can be seen in Figure 2b, where we can see little changes in the interaction strength for carrier phonons. Generally, the thickness dependence phonon scattering plays a vital role in carrier dynamics such as graphene. Similarly, here in ZrTe_3_ nanoparticles thickness reduces the carrier interactions. These results have quite a resemblance with reference [25]. In Figure 2c two-exponential function has been used to fit the dynamic curves (∆A = A_1_e^t^/τ_1_ + A_2_ e^t^/τ_2_), where A_1_ and A_2_ demonstrate the amplitudes of component, ‘t’ display the delay time between the pump-probe light, and τ_1_ and τ_2_ correspond to the material lifetimes [26]. It has been observed that all fitting results display slow and fast components in the process of carrier transfer for all probe wavelengths of ZrTe_3_ NTS. The value of the fast component (τ_1_ ≈ 10 ps) in the conduction band is due to carrier relaxation or the carrier gets trapped due to surface states or localized defects. The value of the slow component (τ_1_ ≈ 140 ps) is due to the recombination of surface state and localized defects processes.

The nanoparticles with different sizes of ZrTe_3_ NTs have different lifetimes trends. The lifetime declines for long wavelengths (>680 nm) in both components as size decreases at long wavelengths. Whereas during a short wavelength of <680 nm, independent decay carrier characteristics have been observed. This phenomenon is due to the difference between surface-bound excitons and in-plane excitons [27]. The transition to PIA (photo-induced absorption) from PIB (photo-induced bleaching) is observed at a probe wavelength of 480–740 nm, which becomes distinguished approximately at the wavelength of 500 nm.

**Figure 3:**
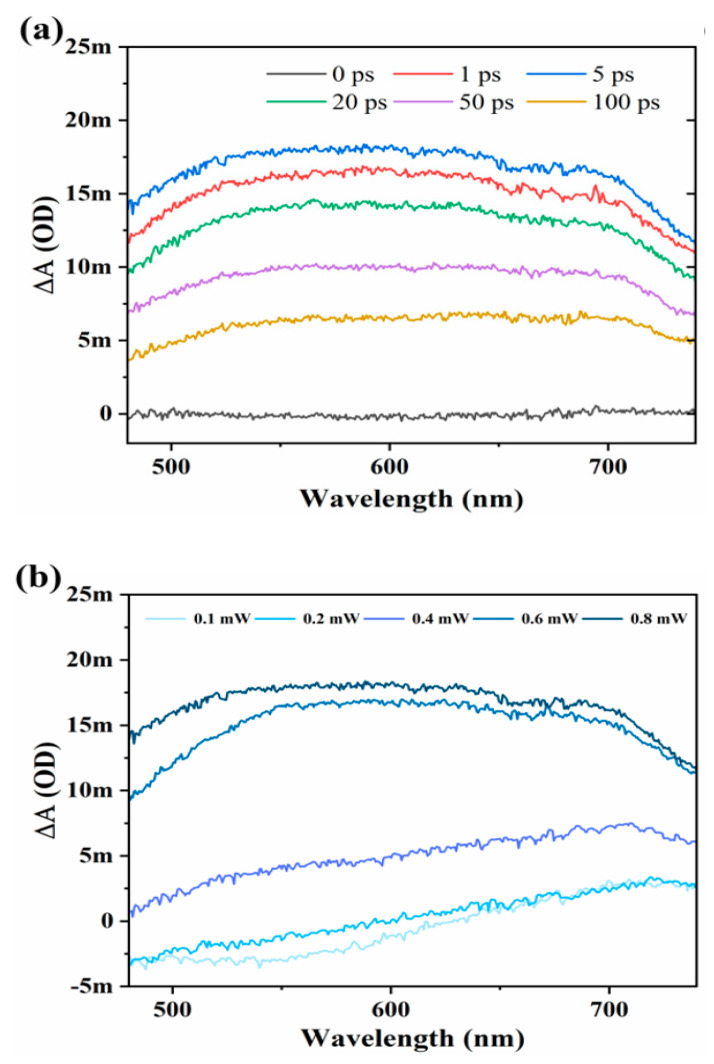
(**a**) Plot of TA versus wavelength at different time delays (0, 1, 2, 5, 20, 50, 100) ps. The first quick charge accumulation in ZrTe_3_ NTS declines by eventual recombination. (**b**) Plot of TA versus wavelength at a different pump power of (0.2, 0.4, 0.6, 0.8) mW. Both (**a**,**b**) TA spectra at selective delay times and selective pump powers are fitted by exponential function for ZrTe_3_ NTS.

To observe the ultrafast TA optical response in ZrTe_3_ NTs, fs-resolved TA spectroscopy is employed. It gives insight information of the carrier’s dynamical process after photoexcitation. The carrier lifetime, cooling dynamics, carrier diffusion, and carrier excitations properties have significant importance to design the high performance and understand the working mechanism in optoelectronics devices. The TA spectra of ZrTe_3_ NTs versus delay line and probe wavelength are shown in Figure 3. From spectra, it can be seen that absorption intensity shows wavelength-type characters, with non-symmetric profiles having a peak at ~600 nm analogous to the energy band-gap. Here it is also observed that decay dynamics is fast for TA spectra is quick initially and slows down later at the delay line of 0.0–100 ps as shown in Figure 3a, which constitutes of several relaxation processes. During the photon, excitation electrons get excited in ultrafast time of 10–100 fs [28], after that many decay dynamics occurred in the cooling process. By scattering, interaction carriers balanced themselves in a short period of 100 fs and formed Dirac Fermi distribution [29]. Phonon-carrier scattering has several ps periods, whereas phonon-phonon scattering has a wide time duration of about 0–10 ps.

Figure 2a demonstrates TA colorful spectra, which display photo-induced absorption (PIA). The absorption enhanced with the increase of pump power, which caused a great number of carriers in the excited state than in the ground state [30]. It has been also observed that signals of PIA decay slower in ZrTe_3_ NTs, which has been further confirmed in the Figure 3a,b these results have a resemblance with reference [31].

**Figure 4:**
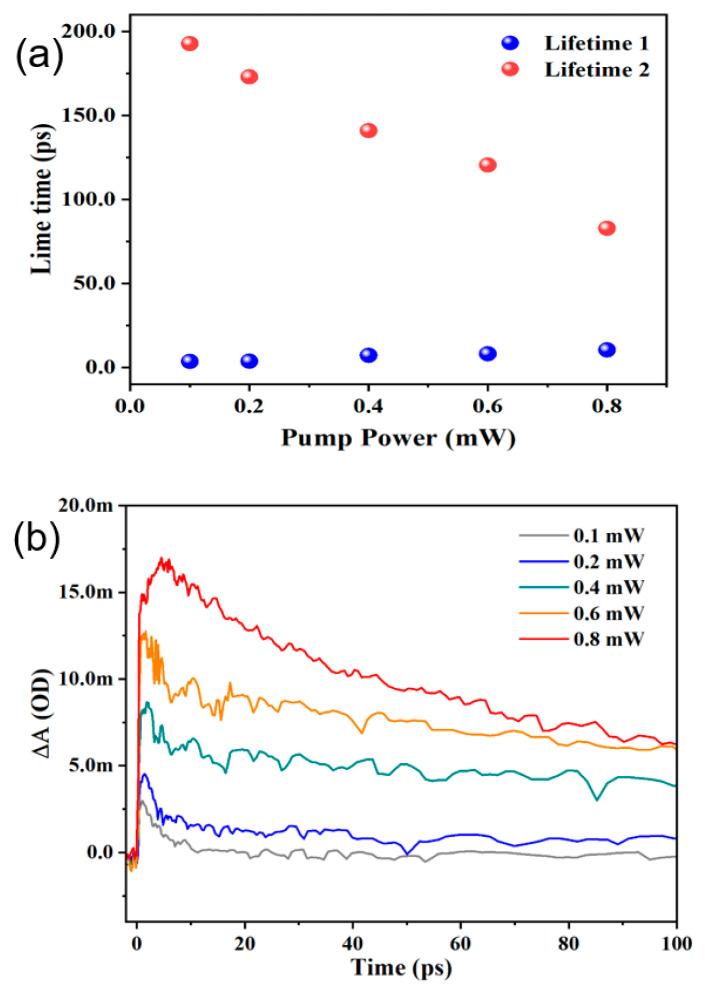
(**a**) Power-dependent carrier dynamics at the probe wavelength of 620 nm; (**b**) the kinetic curves for the carrier kinetic lifetime at each power.

The dynamic curves for different delay times and at different pump powers are shown in Figure 4a,b. At pump power 0.2 mW to 0.4 mW, the ground state bleaching (GSB) and at all delay times (0, 1, 5, 20, 100) ps narrow band gap is showing for ZrTe_3_ NTs. However, the signals of GSB were covered rapidly due to sturdy ESA signals. As compared to 0.2 mW to 0.4 mW, ZrTe_3_ has a larger bandgap at 0.6 mW, which also has GSB and ESA signals. Whereas at 0.8 mW, ZrTe_3_ NTs have pure signals at all delay times. The migration to ESA from GBS further illustrates that TA signals have a broad perspective involving several carrier transfer phenomena. These dynamics processes rely upon pump power and the size of the samples [27].

To understand the power-dependent carrier dynamics in detail, we present in Figure 4b the pump power-dependent dynamic curves at the probe wavelength of 620 nm. It is easy to see that with increasing pump power (0.1–0.8 mW), the kinetic curves are nearly identical. It is worth noting that when the pump power increases, the carrier recombination lifetime gradually decreases, which may be attributed to the existence of nonlinear effects such as Auger recombination at higher pump powers.

The different phenomena of the electron-holes like recombination, trapping and excitations are attributed to charge carrier dynamics. As photo excitation electrons are moved towards higher excited states and holes remain left in valence band. The carrier ‘‘decay’’ dynamics for semiconductors are quite essential to understand the mechanism and its potentials for optoelectronic devices applications. A three-exponential model ΔA = A1$\exp^{t/\tau_{1}}$ + A2$\exp^{t/\tau_{2}}$ + A3$\exp^{t/\tau_{3}}$ has been used to fit the charge carrier decay dynamics.

The ultrafast pump-probe studies on ZrTe_3_ NTS are the same for all TA signals. The TA signals consist of trap carriers and (electron-hole) constituents, which can be increased by TA changes that rely on photoexcitation and properties of carrier lensing owed to spatially localized excitations. The confined spot photoexcitation and clouds scattering create direct imagining for charge clouds. These TA contrasts give a sound proof of quick diffusion and a combination of free charge carriers, which follow bound carrier migration on small scale [32].

**Figure 5:**
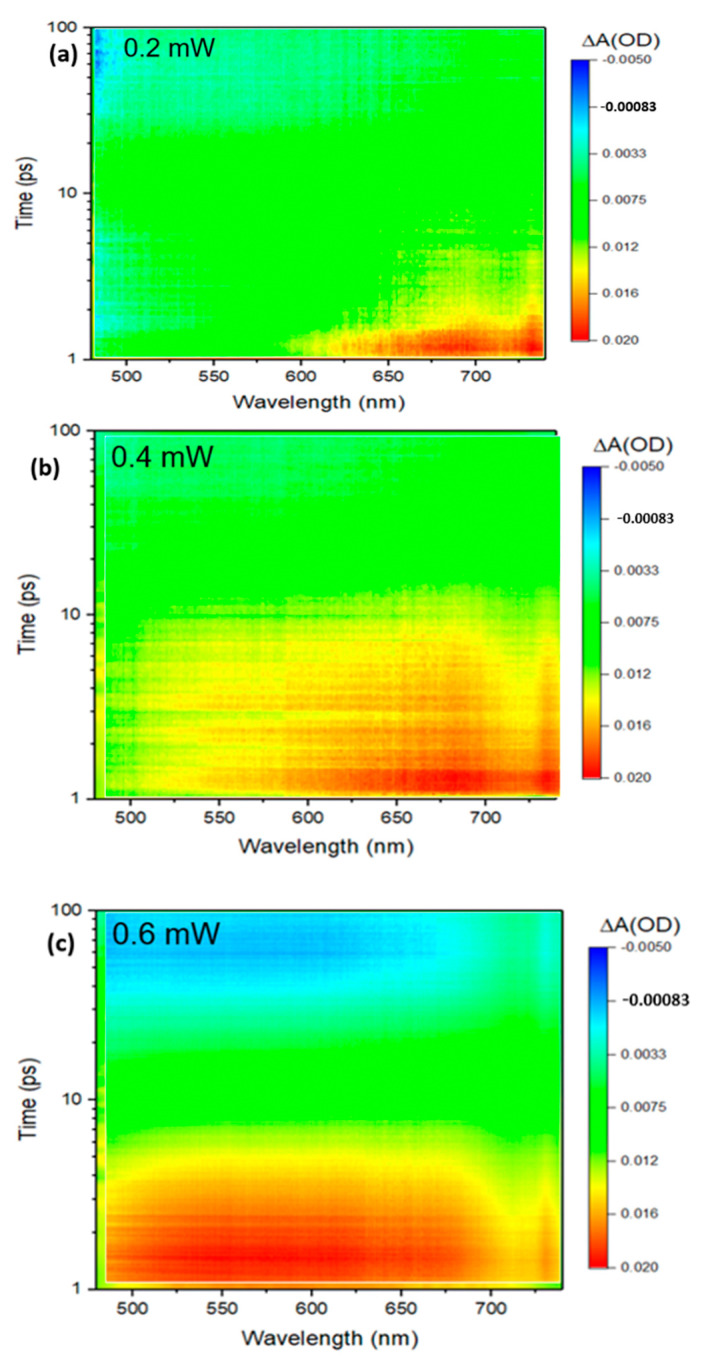

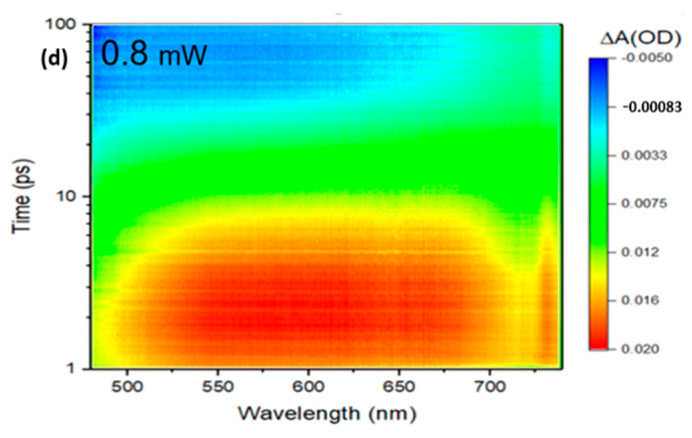
(**a**–**d**) Spectrally and temporally resolved TA signals at pump power (0.2, 0.4, 0.6, and 0.8) mW of ZrTe_3_ NTS. The pump wavelength is 400 nm, and the probe wavelength range is (475–780) nm.

The TA colorful spectra demonstrated in Figure 5a–d display wide band PIA (photoinduced absorption), the absorption increases with an increase in pump power, which shows larger absorption for the excited state as compared to the ground state. The signals of PIA slowly decline in thicker ZrTe_3_ NTS which can be seen in Figure 3a,b for further confirmation [31]. The TA signals for ZrTe_3_ NTS spectrally (475–780 nm) and temporally (1–100 ps) have been assembled at different pump powers of (0.2 mW, 0.4 mW, 0.6 mW, and 0.8 mW) with the help of TA spectrometer and the result has been shown in Figure 5a,d. Here we considered wavelength 400 nm with different pump powers (0.2 mW, 0.4 mW, 0.6 mW, and 0.8 mW). The intensity of pump light is much higher as compared to probe light intensity. Thus, for excited state carriers probe light plays a worthless role [27]. At 0.2 mW ZrTe_3_ NTS shows small TA with fast decay and at 0.4 mW ZrTe_3_ NTS also exhibits wide TA but with little fast decay. As we increased the pump power from 0.6 mW to 0.8 mW for ZrTe_3_ NTS has the highest TA with a slow decline. In Figure 2 the red color peak along the x-axis shows the sturdy absorption region representing the existence of excitons, which demonstrates broad TA with fast decay at a pump power of 0.8 mW. This result indicates potential applications in photoelectric devices [27].

## 4. Conclusions

In this research article, we investigated the TA initial decay in ZrTe_3_ NTS, which exhibits inter-band excitations, as a result, excitons formed due to the interaction of electron-hole pairs. The following evidence support this conclusion, during direct injection of excitons initial decay is usually absent. Secondly, this decay caused the overall decline of signals, which corresponds to the exciton-exciton or electron-hole pairs belonging to exciton resonance in TA.

The study of ZrTe_3_ carrier dynamics is rarely reported, our study provides a basic and detailed understanding of ZrTe_3_ carrier dynamics, which is critical for the design and optimization of ZrTe_3_ based optoelectronic devices significance. We can find other materials, such as PbSe, GeSe [33,34] whose transient absorption studies demonstrate that the relaxation times of tens of picoseconds are suitable for photodetectors. Our ZrTe_3_ has a comparable lifetime and thus also has great potential for application in photodetectors.

The TA spectrometer is an important tool for studying energy level structure and energy relaxation or charge transfer of excited state and emergence of excitons. Which makes the TA spectrometer the most suitable tool to study excitons, excited state dynamics, and detailed absorption spectrum information. The ultrafast carrier dynamics of ZrTe_3_ at different probe wavelengths (480, 540, 600, 680, 740) nm, at different pump powers (0.2, 0.4, 0.6, 0.8) and at different time delays (0, 1, 2, 5, 20, 50, 100) ps was investigated. TA signals approach zero value during a long delay time, which caused an extended lifetime for recombination of carrier relaxation. The lifetime declines for long wavelengths, whereas it is extended during short-wavelength due to the difference between surface-bound excitons and in-plane excitons. At higher pump power the number of carriers (excitons) at the excited state are higher compared to the ground state. The TA was observed to be small with fast decay and at 0.8 mW, results showed the highest TA with a slow decline for ZrTe_3_. As carrier lifetime, cooling dynamics, carrier diffusion, and carrier excitations properties of ZrTe_3_ have significant importance to designing the high-performance and understanding the working mechanism in optoelectronics devices.

## Data Availability

Data supporting reported results can be found by contacting the corresponding author which is responsible of the conducted research.

## References

[1] Mak K.F., Shan J.J.N.P. (2016). Photonics and optoelectronics of 2D semiconductor transition metal dichalcogenides. Nature.

[2] Prodan A., Marinković V., Jug N., van Midden H., Böhm H., Boswell F., Bennett J. (2001). The surface structure and charge distribution of ZrSe_3_ and ZrTe_3_. Surf. Sci..

[3] Geremew A., Bloodgood M.A., Aytan E., Woo B.W.K., Corber S.R., Liu G., Bozhilov K., Salguero T.T., Rumyantsev S., Rao M.P. (2018). Current carrying capacity of quasi-1D ZrTe 3 van der Waals nanoribbons. IEEE Electron Device Lett..

[4] Morozova N.V., Korobeinikov I.V., Kurochka K.V., Titov A.N., Ovsyannikov S.V. (2018). Thermoelectric properties of compressed titanium and zirconium trichalcogenides. J. Phys. Chem. C.

[5] Seshadri R., Suard E., Felser C., Finckh E.W., Maignan A., Tremel W. (1998). The 63 K phase transition of ZrTe 3: A neutron diffraction study. J. Mater. Chem..

[6] Yamaya K., Takayanagi S., Tanda S. (2012). Mixed bulk-filament nature in superconductivity of the charge-density-wave conductor ZrTe 3. Phys. Rev. B.

[7] Yomo R., Yamaya K., Abliz M., Hedo M., Uwatoko Y. (2005). Pressure effect on competition between charge density wave and superconductivity in ZrTe 3: Appearance of pressure-induced reentrant superconductivity. Phys. Rev. B.

[8] Takahashi S., Sambongi T., Brill J., Roark W. (1984). Transport and elastic anomalies in ZrTe_3_. Solid State Commun..

[9] Hoesch M., Bosak A., Chernyshov D., Berger H., Krisch M. (2009). Giant Kohn anomaly and the phase transition in charge density wave ZrTe 3. Phys. Rev. Lett..

[10] Gleason S., Gim Y., Byrum T., Kogar A., Abbamonte P., Fradkin E., MacDougall G.J., Van Harlingen D.J., Zhu X., Petrovic C. (2015). Structural contributions to the pressure-tuned charge-density-wave to superconductor transition in ZrTe 3: Raman scattering studies. Phys. Rev. B.

[11] Ganose A.M., Gannon L., Fabrizi F., Nowell H., Barnett S.A., Lei H., Zhu X., Petrovic C., Scanlon D.O., Hoesch M. (2018). Local corrugation and persistent charge density wave in ZrTe 3 with Ni intercalation. Phys. Rev. B.

[12] Yokoya T., Kiss T., Chainani A., Shin S., Yamaya K. (2005). Role of charge-density-wave fluctuations on the spectral function in a metallic charge-density-wave system. Phys. Rev. B.

[13] Hoesch M., Cui X., Shimada K., Battaglia C., Fujimori S.I., Berger H. (2009). Splitting in the Fermi surface of ZrTe 3: A surface charge density wave system. Phys. Rev. B.

[14] Li M., Dai J., Zeng X.C. (2015). Tuning the electronic properties of transition-metal trichalcogenides via tensile strain. Nanoscale.

[15] Wang X., Chen X., Zhou Y., Park C., An C., Zhou Y., Zhang R., Gu C., Yang W., Yang Z. (2017). Pressure-induced iso-structural phase transition and metallization in WSe 2. Sci. Rep..

[16] Shen P., Ma X., Guan Z., Li Q., Zhang H., Liu R., Liu B., Yang X., Dong Q., Cui T. (2017). Linear tunability of the band gap and two-dimensional (2D) to three-dimensional (3D) isostructural transition in WSe2 under high pressure. J. Phys. Chem. C.

[17] Dybała F., Polak M.P., Kopaczek J., Scharoch P., Wu K., Tongay S., Kudrawiec R. (2016). Pressure coefficients for direct optical transitions in MoS 2, MoSe 2, WS 2, and WSe 2 crystals and semiconductor to metal transitions. Sci. Rep..

[18] Nguyen T.S., Koh J.H., Lefelhocz S., Parkhill J. (2016). Black-box, real-time simulations of transient absorption spectroscopy. J. Phys. Chem. Lett..

[19] Wächtler M., Kupfer S., Guthmuller J., Rau S., González L., Dietzek B. (2012). Structural control of photoinduced dynamics in 4 H-imidazole-ruthenium dyes. J. Phys. Chem. C.

[20] Siebert R., Akimov D., Schmitt M., Winter A., Schubert U.S., Dietzek B., Popp J. (2009). Spectroscopic Investigation of the Ultrafast Photoinduced Dynamics in p-Conjugated Terpyridines. ChemPhysChem.

[21] Bonin J., Costentin C., Robert M., Routier M., Savéant J.M. (2013). Proton-coupled electron transfers: pH-dependent driving forces? Fundamentals and artifacts. J. Am. Chem. Soc..

[22] Changenet-Barret P., Plaza P., Martin M.M., Chosrowjan H., Taniguchi S., Mataga N., Imamoto Y., Kataoka M. (2009). Structural effects on the ultrafast photoisomerization of photoactive yellow protein. Transient absorption spectroscopy of two point mutants. J. Phys. Chem. C.

[23] Davydova D.Y., de la Cadena A., Akimov D., Dietzek B. (2016). Transient absorption microscopy: Advances in chemical imaging of photoinduced dynamics. Laser Photonics Rev..

[24] Zhang W., Ye Y., Liu C., Zhao Z., Wang J., Han J., Zhao X. (2019). Revealing the effects of defects on ultrafast carrier dynamics of CsPbI3 nanocrystals in glass. J. Phys. Chem. C.

[25] Tao X., Mafi E., Gu Y. (2014). Synthesis and Ultrafast Carrier Dynamics of Single-Crystal Two-Dimensional CuInSe2 Nanosheets. J. Phys. Chem. Lett..

[26] Marchioro A., Teuscher J., Friedrich D., Kunst M., Van De Krol R., Moehl T., Grätzel M., Moser J.-E. (2014). Unravelling the mechanism of photoinduced charge transfer processes in lead iodide perovskite solar cells. Nat. Photonics.

[27] Wang R., Jiang X., Gao S., Zhao J., Zhang F., Huang W., Fan T., Liang W., Li Z., Huang H. (2019). Unveiling the Stimulated Robust Carrier Lifetime of Surface-Bound Excitons and Their Photoresponse in InSe. Adv. Mater. Interfaces.

[28] Chen L., Zhang C., Li L., Wu H., Wang X., Yan S., Shi Y., Xiao M. (2017). Ultrafast carrier dynamics and efficient triplet generation in black phosphorus quantum dots. J. Phys. Chem. C.

[29] Brongersma M.L., Halas N.J., Nordlander P. (2015). Plasmon-induced hot carrier science and technology. Nat. Nanotechnol..

[30] Ruckebusch C., Sliwa M., Pernot P., de Juan A., Tauler R. (2012). Comprehensive data analysis of femtosecond transient absorption spectra: A review. J. Photochem. Photobiol. C Photochem. Rev..

[31] Zhang F., Xu N., Zhao J., Wang Y., Jiang X., Zhang Y., Huang W., Hu L., Tang Y., Xu S. (2020). Quantum confinement-induced enhanced nonlinearity and carrier lifetime modulation in two-dimensional tin sulfide. Nanophotonics.

[32] Gabriel M.M., Kirschbrown J.R., Christesen J.D., Pinion C.W., Zigler D.F., Grumstrup E.M., Mehl B.P., Cating E.E.M., Cahoon J.F., Papanikolas J.M. (2013). Direct imaging of free carrier and trap carrier motion in silicon nanowires by spatially-separated femtosecond pump–probe microscopy. Nano Lett..

[33] Gao L., Wang R., Kuklin A.V., Zhang H., Ågren H. (2021). PbSe nanocrystals produced by facile liquid phase exfoliation for efficient UV–Vis photodetectors. Adv. Funct. Mater..

[34] Ma D., Zhao J., Wang R., Xing C., Li Z., Huang W., Jiang X., Guo Z., Luo Z., Li Y. (2019). Ultrathin GeSe nanosheets: From systematic synthesis to studies of carrier dynamics and applications for a high-performance UV–Vis photodetector. ACS Appl. Mater. Interfaces.

